# The persistent pool of HIV-1-infected cells is formed episodically during untreated infection

**DOI:** 10.1128/jvi.00979-24

**Published:** 2024-12-26

**Authors:** Olivia D. Council, Lynn Tyers, Matthew Moeser, Amy Sondgeroth, Ean Spielvogel, Brian D. Richardson, Deelan Doolabh, Shuntai Zhou, Ann Emery, Nancie M. Archin, Bonnie Shook-Sa, David M. Margolis, Salim S. Abdool Karim, Sergei Kosakovsky Pond, Nigel Garrett, Melissa-Rose Abrahams, Sarah B. Joseph, Carolyn Williamson, Ronald Swanstrom

**Affiliations:** 1 Department of Microbiology and Immunology, University of North Carolina at Chapel Hill 318275 , Chapel Hill, North Carolina, USA; 2 Division of Medical Virology, Institute of Infectious Diseases and Molecular Medicine, University of Cape Town 71985 , Rondebosch, Western Cape, South Africa; 3 Lineberger Comprehensive Cancer Center, University of North Carolina at Chapel Hill 169113 , Chapel Hill, North Carolina, USA; 4 Department of Biostatistics, University of North Carolina at Chapel Hill 248512 , Chapel Hill, North Carolina, USA; 5 UNC HIV Cure Center and Department of Medicine, University of North Carolina at Chapel Hill 214908 , Chapel Hill, North Carolina, USA; 6 Center for the AIDS Programme of Research in South Africa (CAPRISA), University of KwaZulu-Natal 56394 , Durban, KwaZulu-Natal, South Africa; 7 Department of Epidemiology, Columbia University Mailman School of Public Health 33638 , New York, New York, USA; 8 Institute for Genomics and Evolutionary Medicine, Temple University 6558 , Philadelphia, Pennsylvania, USA; 9 Division of Public Health Medicine, School of Nursing and Public Health, University of KwaZulu-Natal 296398 , Durban, KwaZulu-Natal, South Africa; 10 National Health Laboratory Services of South Africa 70685 , Johannesburg, Gauteng, South Africa; 11 Department of Biochemistry and Biophysics, University of North Carolina at Chapel Hill 196289 , Chapel Hill, North Carolina, USA; The Ohio State University, Columbus, Ohio, USA

**Keywords:** HIV-1, reservoir, sequencing

## Abstract

Previous studies have shown that the majority of long-lived cells harboring persistent HIV-1 proviral genomes originates from viruses circulating in the year prior to antiretroviral therapy (ART) initiation, but a smaller proportion originates from viruses circulating much earlier in untreated infection. These observations suggest that discrete biological factors influence the entry and persistence of viruses into the persistent proviral pool, and there may be periods earlier in untreated infection with increased seeding. Therefore, we examined the timing of formation of the long-lived pool of infected cells that persists during ART in seven women (after a median of 5.1 years of suppressive ART) by comparing the phylogenetic distance between unique 3′ half genome on-ART proviral sequences and longitudinally sampled pre-ART viral RNA sequences, focusing on the period >1 year prior to ART initiation (i.e., the “early” proviral pool). We constructed models of continuous entry into the persistent proviral pool prior to ART initiation and analyzed the fit of our experimentally derived data to these models. We found that the pattern of persistent proviral pool formation in five of seven participants is incongruent with a model of continuous entry, implying that persistent proviral pool formation can occur episodically during untreated infection. Notably, increased entry into the persistent proviral pool was not universally observed during acute infection, and the timing of enhanced early entry differed across the participants.

**IMPORTANCE:**

Cells harboring HIV-1 proviruses that persist on antiretroviral therapy (ART) constitute the main barrier to an HIV-1 cure. Recent work has elucidated that the majority of persisting proviruses harbor HIV-1 variants circulating near the time of ART initiation, whether the proviruses are intact or defective, though a portion forms earlier in untreated infection. We examined the formation of the “early-forming” persistent proviral pool and found that in 5/7 participants, persistent proviral pool formation was episodic, rather than continuous, suggesting that there are host/biological factors that periodically enhance the formation of the persistent proviral pool. Further characterization of these factors will aid in the development of methods to abrogate their effect, thereby reducing the size of the persistent proviral pool.

## INTRODUCTION

The persistent pool of HIV-1 proviral DNA that persists during successful antiretroviral therapy (ART) was first identified as inducible, replication-competent virus in resting CD4+ T cells (1–3). One theory as to how the persistent proviral pool is formed is that an effector CD4+ T cell, responding to any number of antigens, becomes infected at the time the cell is transitioning to a long-lived memory cell (4). Analyses of integration sites have shown that infected cells in the persistent proviral pool can clonally expand and also transition between different states of differentiation (5–9), thereby maintaining the persistent proviral pool. As such, each integrated copy of proviral DNA is a marker for a single cell that undergoes expansion, contraction, and transitions as part of normal cell function over a period of years, potentially driven by antigenic specificity of the cells and exposure to that antigen (8, 10–13)

The persistent proviral pool consists of cells harboring HIV-1 genomes of varying intactness including inducible outgrowth virus in cell culture, intact proviral DNA, and total proviral DNA, most of which is defective. Inducible outgrowth virus represents only a small fraction of the intact proviral DNA reservoir, and little is known about the subset of intact viral DNA copies that give rise to rebound virus if therapy is stopped. Recent studies have examined when the persistent proviral pool forms (14–21) by comparing viral sequences sampled at different time points pre-therapy to the viral sequences that persist during therapy. Initial reports for proviral DNA (most of which is defective) (14) and for infectious virus in the long-lived reservoir (induced to grow in culture) (15) both showed that, on average, a majority of proviruses that had persisted after years on therapy represents viruses circulating within the last year prior to therapy initiation. This observation has been replicated in several cohorts, comprising multiple HIV-1 subtypes and both male and female participants (16, 17, 21). In addition, a direct comparison of proviral DNA, hypermutated proviral DNA, and inducible infectious virus in the long-lived reservoir showed that all of these forms of proviral sequences entered the persistent proviral pool at similar times, with an increase in persistent proviral pool formation observed around the time of antiretroviral therapy initiation (22). This finding supports the idea that the formation of the pool of persistently infected cells is a phenomenon driven largely by cellular events—that viral DNAs are largely passengers in this process, especially those that are not expressed and that do not disrupt the expression of the gene into which they are inserted.

For people who are untreated for multiple years prior to starting therapy, much of their persistent proviral pool forms late in untreated infection (defined here as within the year of therapy initiation), while a separate portion forms early (i.e., more than a year before the initiation of therapy). Thus, for each of these people, it is possible to estimate the early:late proviral DNA ratio. The early proviral pool must form during ongoing viral replication, while the late proviral pool forms with the attendant blockade of new infection and the dampening of immune activation that antiretroviral therapy provides. Here, we consider two different ways whereby the early proviral DNA pool could form: continuously or episodically. In particular, episodic formation necessitates events that periodically enhance the entry of cells into the persistent proviral DNA pool and by extension into what becomes the latent reservoir.

## RESULTS

### Large data sets of on-ART proviral sequences were obtained from women known to have temporally diverse persistent proviral pools

**Fig 1:**
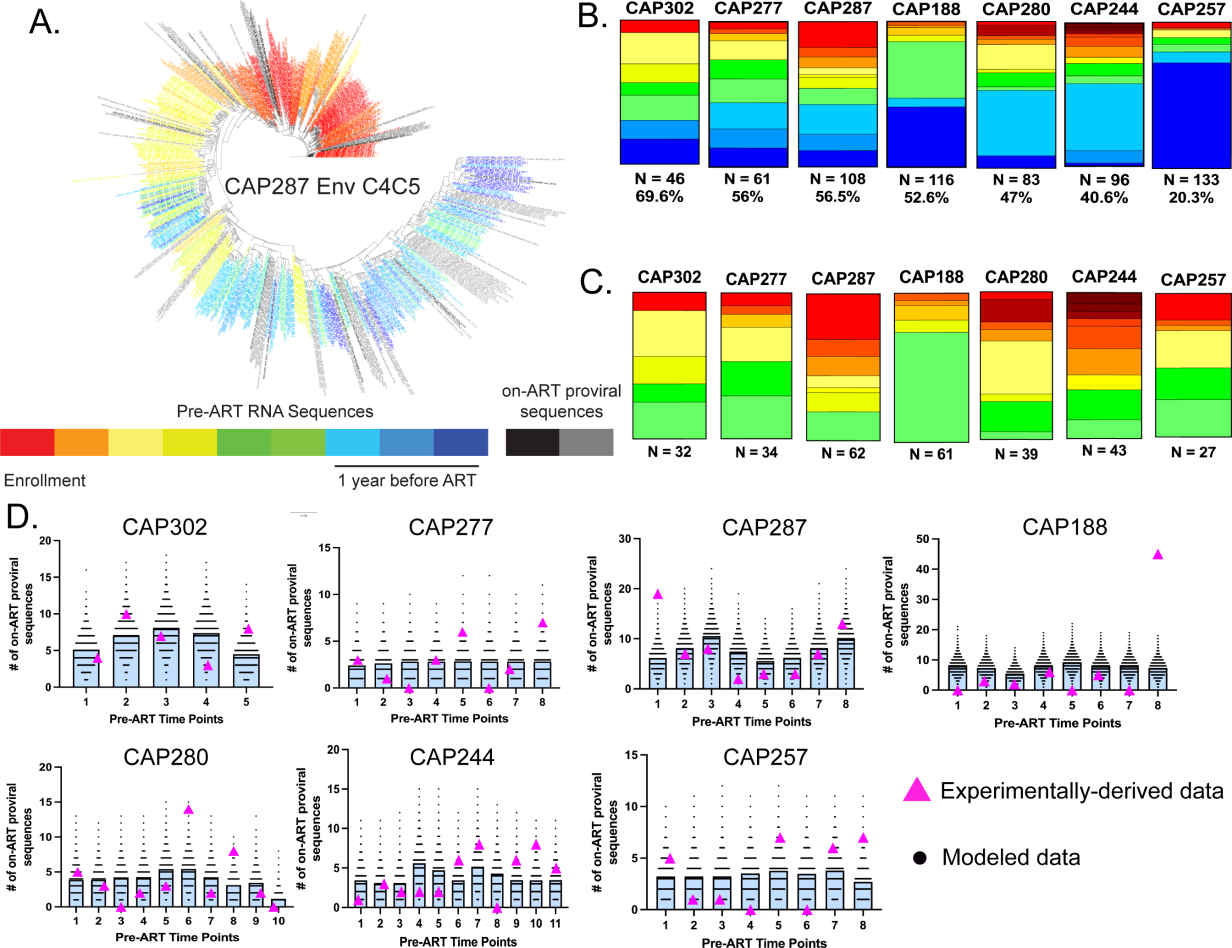
Deep sampling of the early-forming persistent proviral pool and identification of periods of enhanced seeding of the persistent proviral pool. (**A**) A representative approximately maximum-likelihood phylogenetic tree of the C4C5 region of *env* containing pre-ART RNA sequences (colored by collection time point, as illustrated in the panel below), non-hypermutated on-ART proviral DNA sequences (black), and hypermutated on-ART proviral DNA reservoir sequences with hypermutated positions masked (gray). (**B**) Summary of the timing of persistent proviral pool formation for each of the seven participants. The number of unique, on-ART, HIV-1 proviral sequences analyzed, and the percent of unique sequences dating to >1 year before ART initiation (i.e., “early” sequences) is shown below each bar chart. Note: only pre-ART time points for which a corresponding on-ART proviral sequence was obtained are shown in the bar graphs. (**C**) The timing of persistent proviral pool formation when only early sequences are shown. The number of unique “early” (corresponding to >1 year before ART initiation) is shown below each graph. (**D**) Simulated and experimentally-derived distribution of on-ART proviral sequences into each pre-ART bin under the simplified no decay continuous seeding model. Each graph depicts the number of unique on-ART proviral sequences observed at each sampled pre-ART time-point (bin) following 10,000 draws from the “no-decay” model of continuous persistent proviral pool formation (black dots). The number of unique on-ART proviral sequences that were experimentally observed in each pre-ART bin is shown with pink triangles. Bars represent the median.

We re-examined the persistent proviral pool from a subset of seven women on suppressive ART from the larger group previously described in references (15, 22). These women were identified as having a significant fraction (range: 20%–70%, average: 49%; see Table 1) of their persistent proviral pool derived from early sequences, i.e., forming more than a year prior to the initiation of antiviral therapy. We previously obtained between 29 and 46 unique proviral DNA sequences (3' half of the viral genome) per person (22). Here, we have extended the sampling of on-ART proviral sequences to obtain between 38 and 133 genetically unique 3′ half genome sequences from each of these women. As we were primarily interested in examining unique entries into the persistent proviral pool, proviral sequences with fewer than five nucleotide differences were collapsed into a single, representative sequence. The total number of proviruses screened, the percent of proviruses that represent clonal expansion (assessed as being identical sequences), and the total number of unique proviral sequences available for assessment are shown in Table 1. We have included hypermutated sequences by using an approach where presumed APOBEC3G/F mutated sites were masked to allow inclusion with accurate dating of putative entry into the persistent proviral pool (22). We used the longitudinal sequences of viruses in plasma prior to the initiation of therapy (15) as a reference to estimate when the persistent proviral sequences had been replicating prior to the initiation of therapy (Fig. 1A). Briefly, three methods (patristic distance, clade, and phylogenetic placement) were used to estimate the approximate integration date of each proviral sequence based on the phylogenetic relationship to serially collected pre-ART RNA sequences. Each method was used to estimate which sampled timepoint best represented the time when the proviral sequence entered the persistent proviral pool. From these three estimates, a weighted median date was calculated. When the weighted median date was >52 weeks prior to ART, the proviral sequence was considered “early” and was used in the subsequent analyses. Proviral sequences with weighted medians <52 weeks before ART were considered “late” and were excluded from further analyses. In all, 77.4% of proviral sequences dated to a sampled pre-ART time point for this selected subset. When a weighted median estimate was not a sampled timepoint, then the “early” proviral sequence was assigned to the closest sampled pre-ART time point. Per participant, a median of nine proviral sequences (range: 4–14) had weighted medians that were between sampled pre-ART time points and were manually assigned to the closest sampled pre-ART time point. The median distance from the estimated time to the closest sampled pre-ART time point for these proviral sequences was 4.4 weeks (range: 0.02–9.02 weeks).

**TABLE 1:** Summary of proviral reservoir sequences obtained

Participant	Total sequences	No. unique sequences (% clonal)	No. (%) early	No. (%) hypermutated
CAP277	90	38 (58)	22 (58)	16 (42)
CAP280	114	83 (27)	39 (47)	8 (10)
CAP257	152	133 (13)	27 (20)	99 (74)
CAP188	155	116 (25)	61 (53)	16 (14)
CAP244	130	96 (26)	43 (45)	71 (74)
CAP287	131	108 (18)	62 (57)	92 (85)
CAP302	59	46 (22)	32 (70)	31 (67)
Average	119	89 (27)	40 (49)	48 (52)

Longitudinal viral loads are shown for each of the seven participants in Fig. S1. In Fig. 1B, we show the timing of on-ART proviral sequence entry into the persistent proviral pool for each woman. Estimates of the early:late compositions of the persistent proviral pool for these women were not significantly different from our earlier estimates based on smaller numbers of sequences (22) (Fisher’s exact test *P* > 0.4 for all comparisons, Table S1). As we were primarily interested in the seeding dynamics of the early-forming portion of the pool of persistent proviruses, we restricted further analyses to the temporal distribution of the unique proviral sequences that dated to >1 year prior to ART initiation (Fig. 1C).

### The early-forming persistent proviral pool is seeded episodically, not continuously, in the majority of participants in a simplified model of continuous formation

**Fig 2:**
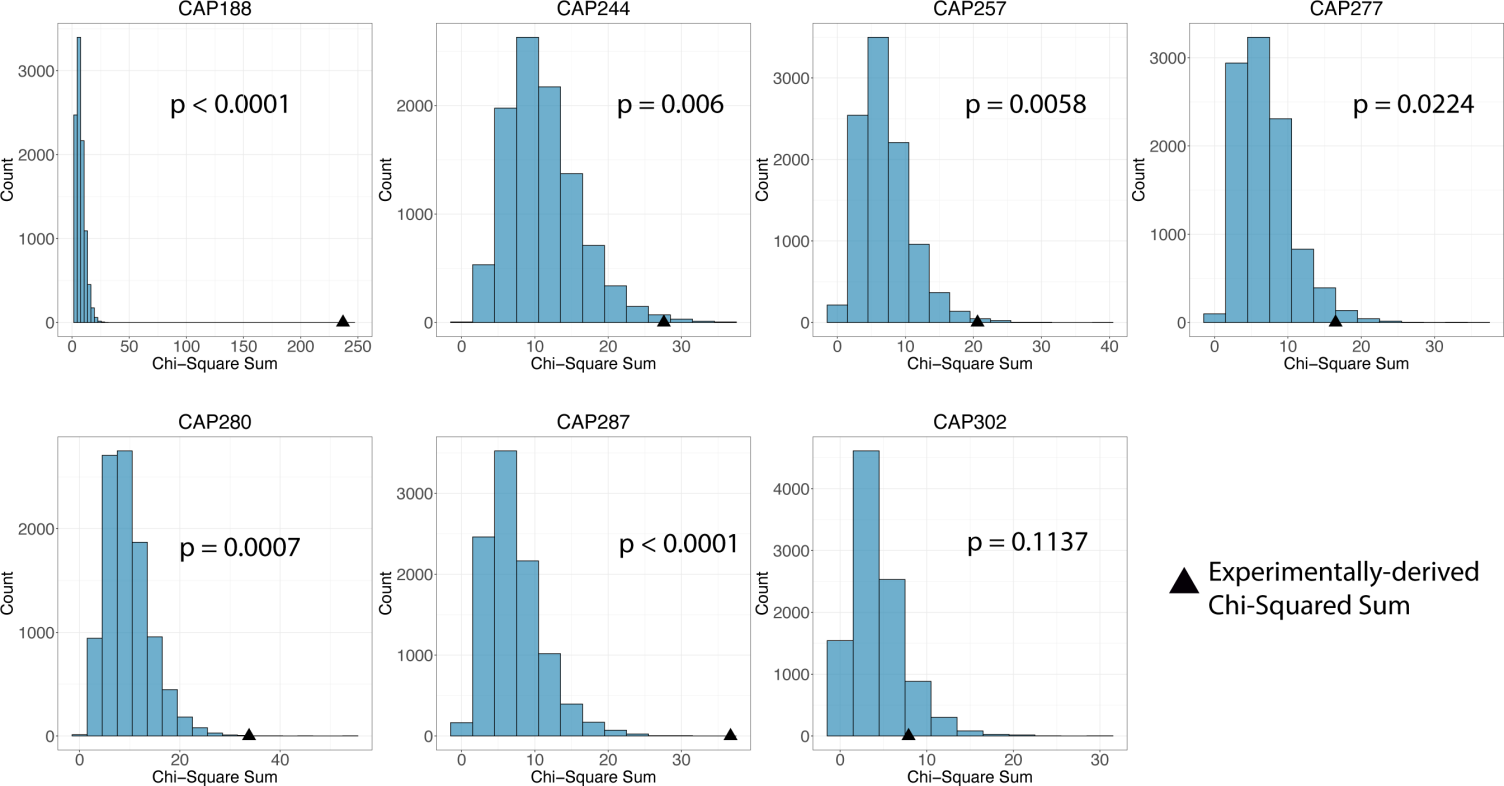
Comparison of the no-decay continuous seeding model to the observed pattern of persistent proviral pool formation. Each graph represents a single participant. *χ*^2^ test statistics are shown on the x-axis, with the count shown on the y-axis. The histogram depicts the distribution of the simulated data’s *χ*^2^ statistic over 10,000 iterations, which are visualized in groups of three in each histogram. Triangles depict the experimentally-derived *χ*^2^ statistic. The *P* values shown represent the proportion of simulated data *χ*^2^ statistics that are greater than or equal to the experimentally-derived *χ*^2^ statistic (*P*-goodness-of-fit).

We tested the hypothesis that the early persistent proviral pool forms continuously during untreated infection. If persistent proviral pool formation were continuous, then the probability that a sampled on-ART proviral sequence would date to a given pre-ART bin is a function of the duration of time encompassed in that pre-ART bin. To match the methodology used in our methods of phylogenetically dating on-ART proviral sequences, we represented each pre-ART time point as the midpoint of each pre-ART bin. The uniform probability of an on-ART proviral sequence dating to each bin is calculated by dividing the number of weeks in the bin by the total duration of “early” untreated infection (from infection to 53 weeks prior to ART). These probabilities are used to weight the random distribution of on-ART proviral sequences into bins using the *random.choices* method from the Random module in Python v3.10.9. Ten thousand draws from this empirical multinomial distribution were used to generate a distribution of per bin sequence counts that would arise under the continuous (constant rate) seeding model. This model is participant-specific, where the number of on-ART proviral sequences being distributed, the number of pre-ART time points to which an on-ART proviral sequence can “date,” and the relative probabilities of an on-ART proviral sequence dating to a given pre-ART bin are specific to the sampling schedule and density of each individual. The simulated data depicting the number of on-ART proviral sequences dating to each pre-ART bin, replicated for 10,000 realizations of the continuous seeding model, are shown in Fig. 1D. For most of the participants, there are certain bins where the experimentally derived values are higher than predicted by almost any of the 10,000 draws from the continuous seeding model (e.g., CAP280 Bin6, CAP188 Bin 8, and CAP287 Bin1). For each simulated data set, we calculated a $\chi^{2}$ test statistic by summing the squared differences between the observed (simulated) number of sequences in each bin and the expected number of sequences in each bin, divided by the expected number of sequences. The expected number of sequences in each bin was defined as N*P, where *N* represents the number of early on-ART proviral sequences, and *P* is the probability of a sequence dating to that bin. This process was repeated for each of the 10,000 simulated data sets for each person. We then generated the same $\chi^{2}$ statistic for the experimentally derived data. A goodness-of-fit *P* value was computed by determining what proportion of the simulated $\chi^{2}$ values was equal to or greater than the experimentally derived value. As seen in Fig. 2, in 6/7 participants, the $\chi^{2\;}$ statistic of the experimentally derived data is greater than 95% of the values generated by the continuous-seeding model (black triangles, *P*-goodness-of-fit < 0.05), indicating that the model for continuous seeding that we have described does not fit the observed pattern of seeding of the persistent proviral pool for most participants. When we apply a Bonferroni correction to the data and consider *P*-goodness-of-fit values < 0.0071 to be indicative of significant deviation from the proposed model, we still find that 5/7 participants have observed patterns of persistent proviral pool seeding that are inconsistent with a continuous seeding model.

### Including pre-ART decay rates in the model of continuous formation only partially explains the observed pattern of reservoir seeding

The inducible, replication-competent reservoir decays with an on-ART half-life of 44 months (1, 23). It is not known if this value is the same for the early and late components of the persistent proviral pool while on therapy, or if a similar decay rate occurs for viral DNA that enters the early-forming persistent proviral pool prior to therapy. Several recent papers have used multiple methods to estimate pre-ART proviral decay (14, 16, 18), reporting half-life estimates ranging from 12 months (14) to 2 years (18). Brooks et al. (18) used a Poisson generalized linear model to infer participant-specific pre-ART proviral half-lives based on the temporal distribution of sampled on-ART proviral sequences. We used the framework of this published model to establish participant-specific pre-ART half-lives for each of our seven participants, using a negative binomial regression model to infer pre-ART proviral half-life from the observed age distribution of the sequenced on-ART proviral genomes. The median pre-ART proviral half-life for these seven women was 1.67 years (range: 0.90–4.6 years) which is similar to previously reported estimates (Table 2). To address the possibility that pre-ART decay of infected cells might affect the chance that a sequence could persist long enough to be sampled, we calculated the percent of the pre-ART persistent proviral pool that would remain following a decay with either a 44-month, a 25-month, or the participant-specific half-life prior to the initiation of therapy and multiplied this by the original probabilities described above and then re-scaled the probabilities. Using a pre-ART proviral half-life of 25 months or 44 months resulted in 4/7 participants having patterns of persistent proviral pool stabilization inconsistent with continuous seeding (*P* < 0.05, Table 3). For two participants, CAP244 and CAP277, the addition of a pre-ART decay rate significantly improved the fit of our original continuous seeding model. When the participant-specific half-lives were used, the model could be rejected (*P* < 0.05) for 6/7 participants (Table 3). Taken together, these results demonstrate that the addition of a pre-ART proviral half-life modestly reduces the number of people in whom we can infer episodic events of early persistent proviral pool formation.

**TABLE 2:** Summary of participant-specific proviral pre-ART half-lives as calculated using a negative binomial regression model.

Participant	Proviral pre-ART half-life, years (months)
CAP188	0.89 (10.7)
CAP244	1.6 (19.2)
CAP257	0.87 (10.4)
CAP277	1.63 (19.6)
CAP280	1.70 (20.4)
CAP287	4.61 (55.3)
CAP302	2.98 (35.8)
Median	1.67 (20)

**TABLE 3:** Summary of *P*-goodness-of-fit values for all continuous seeding models for which the sampled pre-ART time point is the midpoint of the bins (i.e., halfway between the start and end of the bin).^*a*^

Participant	*P*-goodness-of-fit			
	No decay	25-month half-life	44-month half-life	Participant-specific half-life
CAP188	***<0.0001***	***<0.0001***	***<0.0001***	***<0.0001***
CAP244	***0.006***	0.1402	0.0956	**0.017**
CAP257	***0.0058***	**0.0311**	**0.0422**	***0.0008***
CAP277	**0.0224**	0.0617	0.0892	0.0505
CAP280	***0.0007***	***0.0006***	***0.0008***	***0.0007***
CAP287	***<0.0001***	***<0.0001***	***<0.0001***	***<0.0001***
CAP302	0.1137	0.0604	0.1225	***0.0056***

^*a*^ Bolded values are significant at the level of *P* < 0.05, and bolded italicized values are significant when a Bonferroni correction is applied (*P* < 0.0071).

### A second model of continuous formation of the persistent proviral pool also does not fit the observed pattern of formation

The preceding models of continuous seeding of the persistent proviral pool are easy to conceptualize but lack the mathematical elegance and full statistical power that can be realized with a more nuanced model. As such, we explored a different methodology for creating and testing our models of continuous persistent proviral pool formation. To this end, we considered two “null” models, consistent with continuous seeding of the persistent proviral pool: uniform continuous formation (UCF) and continuous formation with decay (CFD). For both models, let $t\;\in \left[0,T_{i}\right]$ measure the time, in weeks, prior to the initiation of ART (i.e., reverse calendar time)*,* where $T_{i}$ is the duration of untreated infection in individual i. Under the UCF model, the probability that the virus circulating at time t enters the persistent proviral pool (and is present at the time of sampling) is constant, i.e., P (t) ∼ C. The CFD model incorporates the participant-specific half-life of infected cells during untreated infection, estimated directly from the temporal distribution of experimentally derived on-ART proviral sequences using maximum likelihood. Under the CFD model, the probability that the virus circulating at time t enters the persistent proviral pool and persists long enough to be sampled is controlled by the individual-specific pre-ART decay rate $\lambda_{i}$, measured in weeks, and is given by $P\;\left(t\right)\;∼\;C\;e^{-t/\lambda_{i}\log\left(2\right)}$.

Because our time measurement resolution is limited to discrete temporal bins, we discretize the two continuous-time models above as follows. For individual i, let $S_{i,k}$, $T_{i}\leq S_{i,D_{i}}<\ldots <\;S_{i,1}\leq 0$, denote the *k*-th of $D_{i}>1$ sampling times for this individual, measured from the initiation of ART, with larger *k* corresponding to older samples. If we further define time boundaries as $S_{i,0}=\;52,\;\;S_{i,D_{i}+1}=\;2T_{i}-S_{i,D_{i}},$ then the catchment window for each time interval k is the time interval between $\left(S_{i,k-1}+S_{i,k}\right)/2$ and $\left(S_{i,k+1}+S_{i,k}\right)/2$, i.e., the span between the midpoints surrounding sampling time *k*. The most recent interval ($k=1D_{i}$) extends to the time of ART initiation less than 1 year, and the least recent interval ($k=D_{i}$) extends to the estimated start of infection. The probability that a sequence enters the persistent proviral pool at time interval d is described as follows for each model of continuous formation:

1. Discretized uniform continuous formation (dUCF): This is a multinomial distribution on $D_{i}$ categories (time bins), where the probability that a sequence from time interval d enters the persistent proviral pool is proportional to the catchment span of the interval, i.e., $P\;\left(interval=d\right)\;∼\;\left(S_{i,k-1}-S_{i,k+1}\right)/\left(T_{i}-52\right)$.

2. Discretized continuous formation with decay (dCFD): This is a multinomial distribution on $D_{i}$ categories (time bins), where the probability that a sequence from time interval d enters the persistent proviral pool is proportional to the integral of the decay probability over the catchment span of the interval, i.e., $P\;\left(interval=d\right)\;∼\;\left(S_{i,k-1}-S_{i,k+1}\right)/\left(T_{i}-52\right)$.

### As seen in the simplified models, the early-forming persistent proviral pool is seeded episodically, not continuously, in the majority of participants

**Fig 3:**
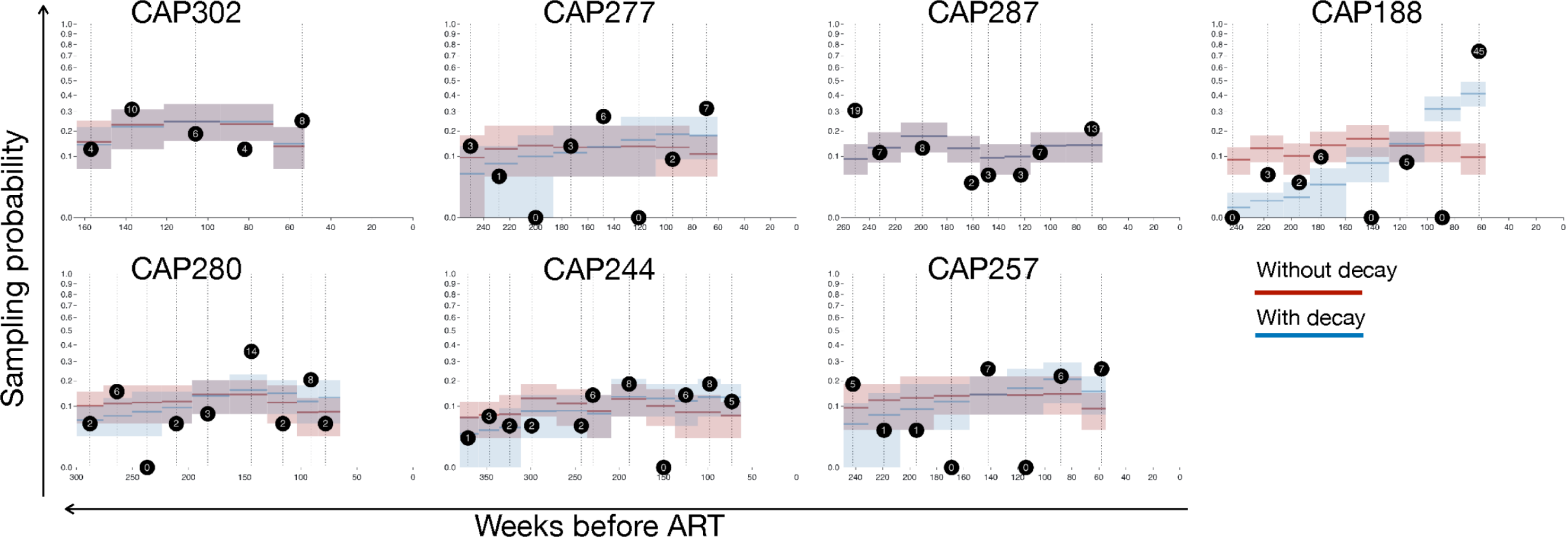
dUCF and dCFD models for each participant. Each graph represents a single participant. The duration of untreated infection is shown on the x-axis. Sampled pre-ART time points are shown as vertical dashed lines. The number of unique on-ART proviral sequences mapped to each pre-ART time point is shown in black circles, while the corresponding fraction (of the total number of unique on-ART proviral sequences analyzed) is shown on the y-axis; these are the maximum likelihood estimates for the multinomial model. The catchment intervals for each sampling time are depicted as horizontal lines (red = dUCF, no pre-ART decay, blue = dCFD, and incorporates pre-ART decay), and the corresponding sampling probabilities for the dUCF and dCFD models are reflected on the y-axis. Shading represents the 10%–90% interquartile range for the 10,000 replicates of each model.

The seeding probabilities, reflecting the likelihood that variants circulating at the specified time points would enter the persistent proviral pool and persist until the time of sampling for each participant under both models, are shown in Fig. 3. As expected, when a pre-ART decay rate is applied, seeding probabilities increase as the time prior to ART decreases (i.e., increased rate of seeding is expected at the latest pre-ART time points). The number of on-ART proviral sequences that date to each pre-ART time point is shown in black circles. Several participants have instances of increased entry into the persistent proviral pool (CAP188: 60 weeks before ART, CAP287: 260 weeks before ART, and CAP280: 150 weeks before ART), with observed seeding rates between two and three times the probabilities given by either continuous seeding model. Notably, and consistent with what was observed in our simplified model, these examples of increased persistent proviral pool formation do not occur at uniform times (e.g., acute infection) across participants, unlike the previously observed increase in persistent proviral pool formation that occurs near the time of ART initiation (15), suggesting that the driving force behind these episodic increases in the persistent proviral pool formation seen at some times during early untreated infection do not correspond to a shared, temporally constrained event.

The fit of each model of continuous persistent proviral pool formation (dUCF and dCFD) was tested as follows. For a given set of sequence counts, in each time bin, the maximum likelihood estimator (MLE) for the parameters of the multinomial distribution is the fraction of counts assigned to the bin. We tested whether the dUCF null model could be rejected (vs the MLE), using the likelihood ratio test (LRT), both with the asymptotic distribution of the test statistic $\chi_{D_{i}-1}^{2}$ and via parametric bootstrap with 10^5^ replicates. To test whether the dCFD model could be rejected, we first fitted the per-individual decay parameter using maximum likelihood and then used the LRT test with the asymptotic distribution of the test statistic $\left(\chi_{D_{i}-1}^{2}\right)$ and via parametric bootstrap with 10^3^ replicates.

**Fig 4:**
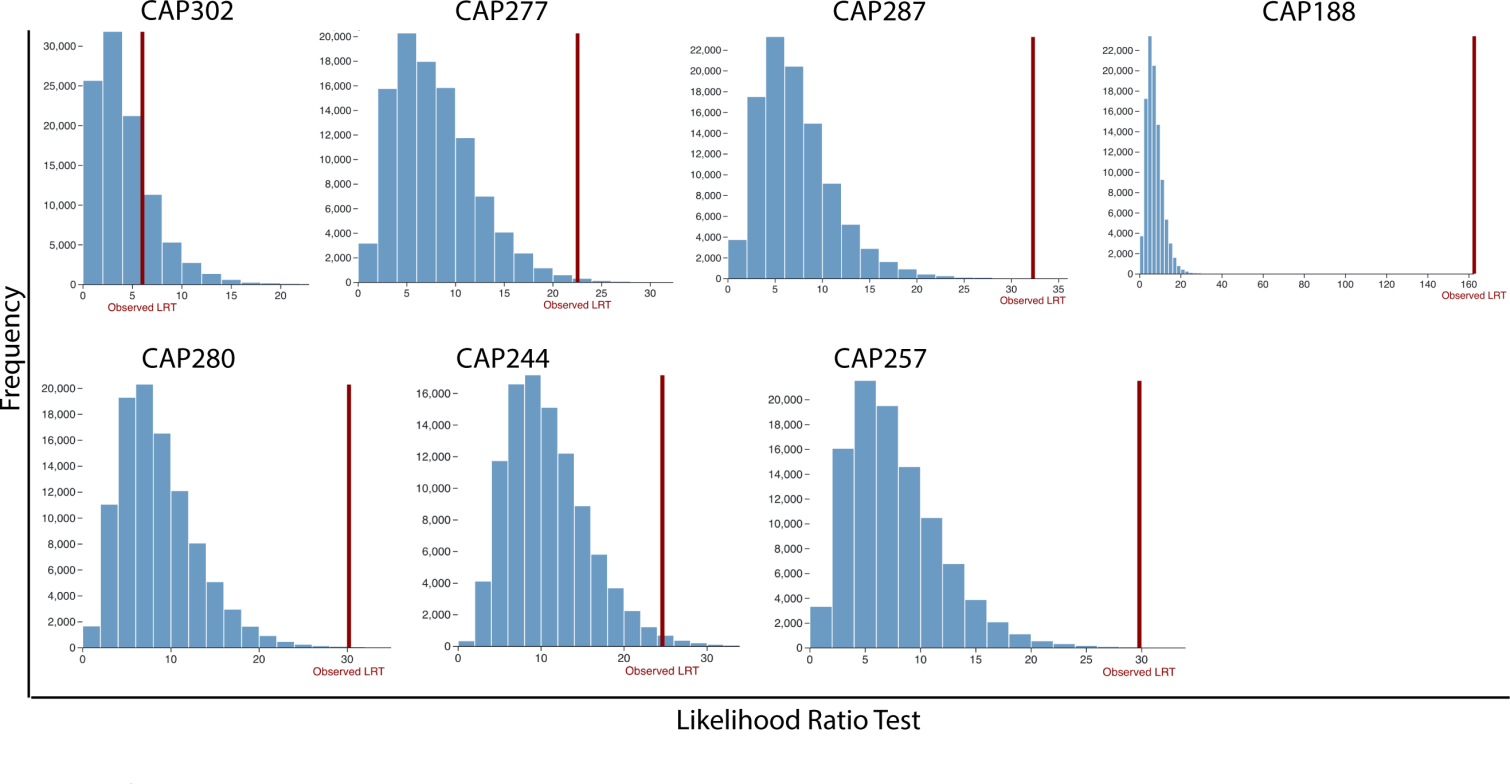
Testing dUCF model fit using LRT. Each participant is shown in a separate plot. The LRT is shown on the x-axis, and the count is shown on the y-axis. The histograms represent the LRT for each of 10^5^ parametric bootstrap replicates of the MLE. The observed LRT is shown in a red line.

In both the uniform continuous formation and continuous formation with decay models, five of seven participants had bootstrap *P* values that were significant at the *P* < 0.05 level after Holm-Bonferroni family-wise error rate correction (i.e., rejecting the model; Fig. 4; Table 4), indicating that the observed pattern of proviral pool formation and persistence does not occur at a uniform rate during untreated infection. While the addition of a pre-ART decay rate did not significantly improve the overall fit of the model, it did increase the *P* values (Table 4), suggesting an improved fit compared to the uniform continuous formation model. However, pre-ART decay rates alone do not explain the observed pattern of proviral pool formation and persistence.

**TABLE 4:** Summary of dUCF and dCFD model fit results^*a*^

Participant	dUCF		dCFD	
	*P*, asymptotic	*P*, bootstrap	*P*, asymptotic	*P*, bootstrap
CAP188	**0**	**1 × 10^−5^**	8.15 × 10^−14^	**0.0001**
CAP287	3.60 × 10^−5^	**7 × 10^−5^**	3.6 × 10^−5^	**0.001**
CAP257	1.55 × 10^−5^	**2 ×** 10–5	9.1 × 10^−5^	**0.001**
CAP280	8.07 × 10^−5^	**0.00017**	0.00030	**0.001**
CAP277	3.31 × 10^−5^	**6.0 × 10^−5^**	0.0024	**0.001**
CAP244	0.0061	0.0111	0.068	0.059
CAP302	0.1988	0.2152	0.2056	0.1707

^*a*^ Bold values are significant at the *P* < 0.05 level after Holm-Bonferroni family-wise error rate correction to bootstrap *P* values.

### Elevated seeding of the persistent proviral pool is not observed during the first year of infection in most participants who initiated ART during chronic infection

**Fig 5:**
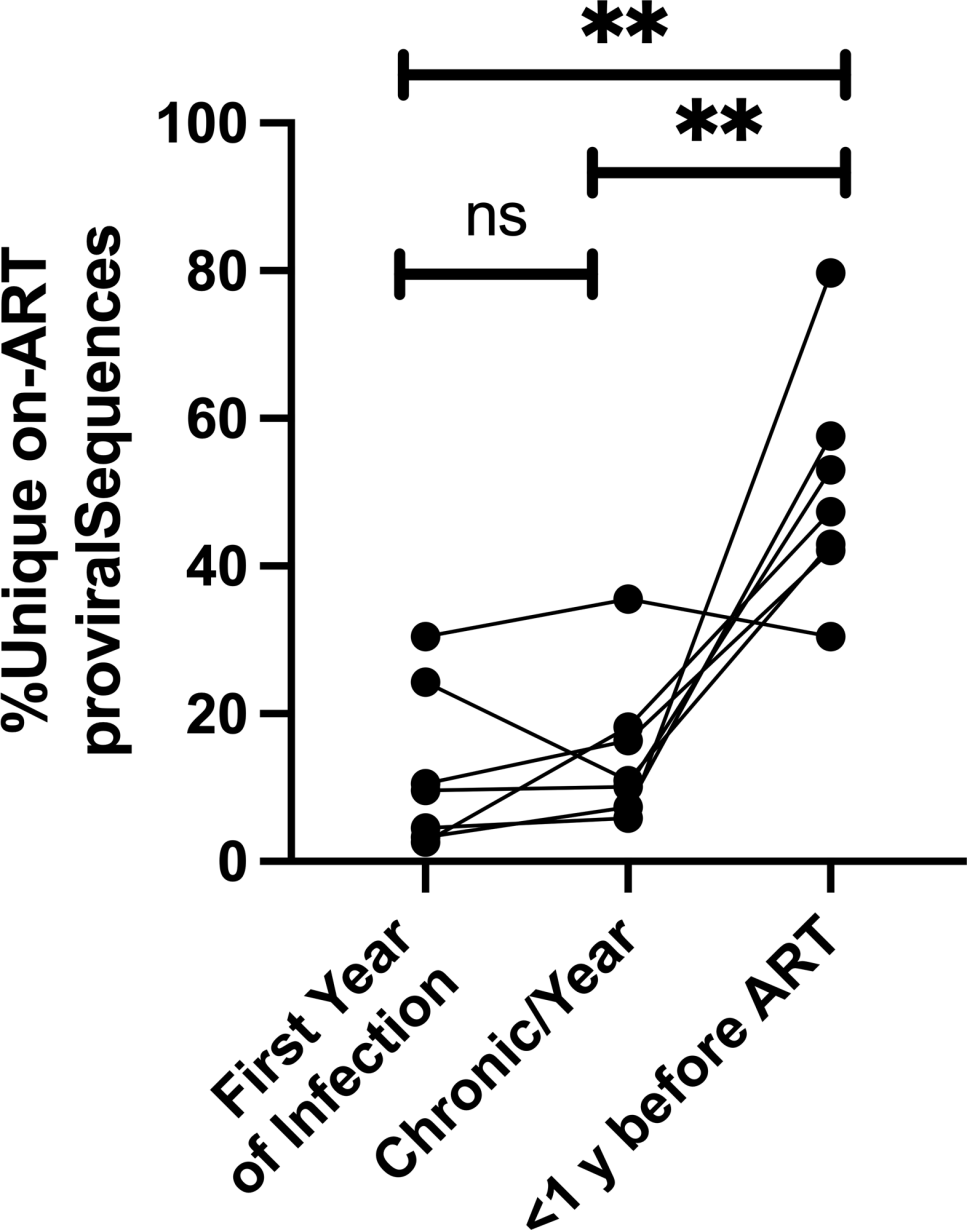
No increase in persistent proviral pool seeding is observed during acute infection. The percent of unique on-ART proviral sequences seeded at each point (first year after transmission, chronic per year, and <1 year before ART) is shown. Paired *t*-tests were used to compare the percent of unique on-ART proviral sequences that were seeded at the indicated times. **P < 0.005 .

We were interested in determining if there was a common event that drove episodic early persistent proviral pool formation across our participants. To this end, we calculated the percent of unique on-ART proviral sequences seeded during (i) the first year of infection, (ii) per year throughout chronic infection, and (iii) in the year before ART initiation (Fig. 5). As expected, there was a statistically significant increase in the percent of unique on-ART proviral sequences seeded in the year before ART compared with either acute infection or chronic infection even in this selected group, again identifying this as a period of enhanced entry into the persistent proviral pool (*P* = 0.008 vs acute and *P* = 0.0042 vs chronic, paired *t* tests). However, we did not observe a significant increase in persistent proviral pool formation during the first year of infection, compared to during chronic infection (paired *t* test). It is important to note that our participants initiated ART during chronic infection. It has previously been observed that for individuals treated during acute infection, the size of the replication-competent reservoir correlates with viral load (24), consistent with our observation that significant entry into the persistent proviral pool can happen during acute infection; however, the first year of infection is the least active time compared to later times during the untreated period for the formation of persistent proviruses that survive a period of untreated infection followed by several years of suppressive therapy. As clones of cells infected during early infection have had the longest to decay, which could impact our ability to adequately sample them, we repeated the analysis described above, where we only included individuals (*n* = 4) for whom the number of unique on-ART proviral sequences that dated to the earliest pre-ART time point was equal to or greater than the expected number of sequences under the no decay continuous seeding model. This model represents the most generous estimate of the number of expected sequences, as it does not consider proviral decay. In restricting the analysis in this way, we are only analyzing individuals for whom we have some evidence that on-ART proviral sequences from the earliest time point are not underrepresented in our sampling. The results from this second analysis, shown in Fig. S2, mirror what we observed with the complete cohort. Specifically, we do not find an increased rate of persistent proviral pool stabilization in the first year of infection compared to either the per-year rate during chronic infection or the year before ART.

## DISCUSSION

Early initiation of ART can limit the size of the pool of infected cells harboring persistent proviral genomes (25–27). However, initiation of ART before detectable viremia in a non-human primate model (28), or during Fiebig stage 1 in humans (29), does not prevent the formation of a persistent proviral pool capable of reigniting infection when ART is stopped. Taken together, these observations have led to the hypothesis that the persistent proviral pool begins forming during acute infection with a substantial portion stabilized later near the time of ART initiation. The dynamics of entry into the persistent proviral pool throughout untreated infection have not been elucidated, particularly as it relates to the portion of the persistent proviral pool seeded during early (>1 year before ART) infection. Here, we used phylogenetic analyses and modeling to examine the hypothesis that the early-forming persistent proviral pool is continuously seeded.

Recent work by Joseph et al. (22) and Kinloch et al. (30) has revealed that the cells harboring intact and defective HIV-1 proviruses enter the persistent proviral pool with similar kinetics, and the majority of variants enter the persistent proviral pool near the time of ART initiation. This can be viewed as an “episode” of persistent proviral pool formation. By increasing the sampling depth in this study and focusing on early-forming variants, we identified additional periods of enhanced entry into the persistent proviral pool not evidently linked to the biological processes occurring as a result of ART initiation. These findings suggest at least two plausible mechanisms of enhanced formation of the persistent proviral pool. (i) Encounters with various antigens (HIV-1 or other transient infections) could facilitate reservoir seeding in several ways. First, antigen-driven clonal expansion increases the pool of susceptible cells, creating a temporal window during which an increased number of CD4+ T cells become infected. During the contraction phase, most of these cells die but some transition into long-lived memory CD4+ T cells. The surviving, HIV-infected memory CD4+ T cells thus contribute to the long-lived reservoir. Similarly, if HIV-infected cells undergo antigen-driven clonal expansion, this would also serve to increase the probability that a clone would contribute to the persistent proviral pool. Furthermore, exposure to antigen leads to an increase in the number of activated CD4+ T cells, which can enhance infection leading to an increased opportunity for the eventual entrance into the persistent proviral pool. (ii) HIV-induced lymphopenia causes an increase in serum levels of IL-7 (31, 32). This drives the homeostatic proliferation of cells to replace the lost lymphocytes (33). Thus, a period of rapid CD4+ T cell loss could be followed by rapid CD4+ T cell restoration, meaning that there would be a larger pool of target cells in a certain temporal window surrounding this event. Some of these infected cells would stochastically enter the persistent proviral pool. Both of these mechanisms involve a period of enhanced transition of CD4+ T cells from effector to long-lived memory phenotype, similar to the proposed mechanism driving the period of enhanced persistent proviral pool formation observed near the time of ART initiation (34). It is also possible that episodic seeding may be driven by changes in viral fitness which occur iteratively over the course of untreated infection in response to immune pressure. If a viral mutant had recently evolved cytotoxic T lymphocyte (CTL) escape mutations and thus was able to infect a greater number of susceptible cells in a temporal window, more infected cells from this period may stochastically transition to latency, which would also appear as an episode of enhanced seeding.

Recently, several models of pre-ART proviral decay rates and persistent proviral pool formation have been proposed (16, 18). Our current study is consistent with some conclusions of these previous studies, notably the lack of an increased period of persistent proviral pool seeding during acute infection in individuals who initiated ART during chronic infection. It is important to note that these earlier studies include analyses of on-ART proviral variants seeded in the year prior to ART initiation. Here, we extend these findings by deeply sampling the early-forming persistent proviral pool and find that for most participants, the observed data are not consistent with a model solely of continuous entry into the pool of persistently infected cells. Our data do not exclude the possibility of low-level continuous seeding of the persistent proviral pool with brief periods of enhanced seeding driven by yet unknown biological phenomena. As such, there may be additional opportunities and mechanisms by which to block the formation of large portions of the persistent proviral pool.

There are important caveats to this study. As this work was performed using on-ART samples, we are inherently studying the surviving clones of cells infected during untreated infection. There may be smaller clones that are less readily sampled that are seeding in a continuous manner, but are below our threshold for detection or were eliminated before the sampled on-ART time point. Importantly, recent work has suggested that the replication-competent and defective persistent proviral pools form with similar kinetics and are equally likely to be part of a group of identical sequences (22). Additionally, since our work focuses on the 3′ half of the genome, we are unable to include proviral variants with complete deletions of Env or Nef, as can occur due to homologous recombination of the LTR, nor can we extrapolate our results to solo LTRs, which may have different characteristics due to their inability to express viral proteins or antigens. These variants may enter the reservoir with different kinetics than those described here.

## MATERIALS AND METHODS

### Study participants

Participants were part of the CAPRISA-002 cohort which enrolled women from rural and urban KwaZulu-Natal, South Africa who were identified during acute/primary HIV-1 infection and followed longitudinally (35).

### Genomic DNA extraction

Total DNA was extracted from total peripheral blood mononuclear cells collected a median of 5.1 years after the initiation of ART using the DNeasy Blood and Tissue Kit (Qiagen, cat # 69504). The purified DNA was eluted in 200 μL of diethylpyrocarbonate (DEPC)-treated water, and the eluate was placed back on the column to elute the remaining DNA. Extracted DNA was stored at −20°C until use.

### Pre-ART RNA sequencing

Longitudinal pre-ART RNA sequencing was performed using MiSeq with Primer ID as previously described (15). Up to four regions of the HIV-1 genome, ENV C1C2 (HXB2 #6,585–6,950), ENV C2C3 (HXB2 #6,839–7,321), ENV C4C5 (HXB2 #7,371–7,685), and NEF1 (HXB2 #8,786–9,201), were used to phylogenetically date on-ART HIV-1 proviral sequences. Pre-ART RNA sequences were deposited in the Sequence Read Archive under accession numbers PRJNA1034561 to PRJNA550394.

### Droplet digital PCR (ddPCR)

ddPCR using primers/probes targeting HIV-1 LTR/*gag* and the human *RPP30* gene was carried out as previously described (36, 37). Briefly, 22 μL reactions were prepared in duplicate for each target and sample. LTR/*gag* reactions consisted of 10 μL ddPCR Supermix for Probes (no dUTP; BioRad, catalog #1863024), 900 nM each of LTRgagF, and LTRgagR, forward and reverse primers, respectively, and 318 nM LTR/*gag* probe (see Table S2 for sequences). A total of 3 μL of undiluted template DNA was added to each reaction, and the remaining reaction volume consisted of diethylpyrocarbonate (DEPC)-treated water (Invitrogen, Catalog # AM9906). *RPP30* reactions were prepared in the same manner, utilizing 900 nM each of RPP30F and RPP30R primers and 318 nM of RPP30 probe. Template DNA for *RPP30* reactions was diluted 1:100 in DEPC-treated water, and 1 μL was added to each ddPCR reaction. No template control wells were included on every plate, as well as positive control wells for LTR/*gag* and *RPP30* amplicons. Droplets were generated using an automated droplet generator (BioRad, Catalog # 1864101). Following droplet generation, plates were subjected to PCR amplification with the following cycling conditions: an initial denaturation at 95°C for 10 min, then 45 cycles of 95°C for 30 seconds and 60°C for 1 min, a final incubation at 98°C for 10 min, and a 4°C hold. Droplets were read on the QX200 Droplet Reader (BioRad) with the following parameters: Experiment = Rare Event Detection, Mix = ddPCR Supermix for Probes (no dUTP), Target 1 = FAM, and Target 2 = VIC. No template control reactions were used to set the thresholds for all reactions of a given target. Results were averaged over replicate reactions.

### HIV-1 DNA half-genome amplification

Amplification of the 3′ half of the HIV-1 proviral DNA genome (HXB2 coordinates: 4,653–9,632) was carried out by nested PCR at limiting dilution. First round PCR reactions had a final volume of 20 μL and consisted of 14.8 μL DEPC-treated water, 2 μL of 10× Buffer (Invitrogen), 2 mM MgSO_4_, 2 mM dNTP Mix (Promega, catalog # PRU1515), 0.4 mM U5-623F forward primer, 0.4 mM U5-601R reverse primer, and one unit of Platinum *Taq* DNA Polymerase High Fidelity (Invitrogen, cat # 11304102). See Table S2 for primer sequences. Template DNA was added such that one copy of HIV-1 DNA was present per PCR reaction. No template controls were included in every plate. The first round of thermal cycling conditions were as follows: an initial denaturation of 92°C for 2 minutes, then 10 cycles of 92°C for 10 seconds, 60°C for 30 seconds, and 68°C for 10 minutes, followed by 20 cycles of 92°C for 10 seconds, 55°C for 30 seconds, and 68°C for 10 minutes. A final 68°C extension was performed for 10 minutes, followed by a 4°C hold. Nested second-round PCR reactions were conducted in a final volume of 50 μL, comprising of 34.8 μL DEPC-treated water, 5 μL 10× Buffer, 2 mM MgSO_4_, 2 mM dNTP mix, and one unit of Platinum *Taq* DNA Polymerase High Fidelity. PacBio barcoded forward (4653F) and reverse (OFM19) primers were added to a final concentration of 0.1 mM, with each reaction receiving a unique barcode. To each reaction, 2 μL of first-round PCR product was added. The second round of thermal cycling conditions were as follows: an initial denaturation at 94°C for 2 min, then 10 cycles of: 94°C for 15 seconds, 55°C for 30 seconds and 68°C for 8 minutes, followed by 25 cycles of: 94°C for 15 seconds, 55°C for 30 seconds, 68°C for 8 min—adding 20 seconds each cycle—and a final extension at 68°C for 7 minutes followed by a 4°C hold. Second-round PCR products were analyzed by gel electrophoresis using a 0.8% agarose gel stained with SYBR safe DNA Gel Stain (Thermo Fisher Scientific) and viewed with a UV gel imager (Carestream).

### PacBio library construction

Appropriately sized amplicons were pooled in equal volumes, and gel was extracted using a 0.8% agarose gel and the MinElute Gel Extraction Kit (Qiagen, catalog # 28604) according to the manufacturer’s instructions with a single modification. Following the PE Buffer step, the flow-through was discarded, and columns were centrifuged at 13,000 rpm for 3 minutes to eliminate any residual ethanol. The DNA concentration of the purified, pooled, barcoded PCR products was determined using the Qubit Broad Range dsDNA kit (Invitrogen, catalog # Q32853). PacBio libraries were constructed using 5 µg of the purified, pooled, barcoded 3′ half-genome HIV-1 PCR amplicons. Library preparation was conducted using the SMRTbell Template Prep Kit 1.0 (PacBio, product discontinued and replaced with the SMRTbell Template Prep Kit 3.0, Pacific Biosciences of California, Inc, catalog # 102–141-700) according to the manufacturer’s instructions. The final sequencing library was purified three sequential times using AMPure PB Beads (PacBio, catalog #100–265-900). PacBio libraries were sequenced at North Carolina State University’s Genomic Sciences Laboratory.

### Processing of HIV-1 DNA sequences

Raw sequencing reads from the PacBio instrument were de-multiplexed using LIMA. Participant-specific consensus sequences of near full-length, Quantitative viral outgrowth assay (QVOA)-derived sequences (15) were used as a reference. First, sequences that passed quality control criteria were assembled to the participant-specific QVOA-derived consensus sequence using Geneious and default parameters. Reads that aligned to the 5′ half of the genome were discarded. Next, primer sequences were trimmed from the ends of the reads. Barcodes that returned multiple sequences with more than five nucleotide differences between them were discarded. Neighbor-joining trees were constructed with all 3′ half genome sequences from a given participant to check for the presence of clones. Clones were defined as sequences with different barcodes that had fewer than five nucleotide differences. Clones were enumerated, and a single representative sequence from each clone was retained for further analysis. Next, hypervariable loops were manually trimmed from each sequence. Processed on-ART proviral sequences were examined for the presence of hypermutation using LANL Hypermut 2.0 with a participant-specific, QVOA-derived sequence as the reference. Putatively hypermutated positions in statistically significantly hypermutated on-ART proviral sequences were replaced with “N” (i.e., masked) using an in-house script. We estimated the date of entry into persistent proviral pool for each on-ART proviral DNA sequence using phylogenetic methods as previously described (15). The 3' half genome sequences are deposited in GenBank (accession nos. PP295393 to PP295816).

### Simplified models of continuous reservoir formation

#### Continuous seeding without pre-ART decay

For each participant, the duration of the bin was calculated, and the probability that an on-ART proviral sequence would be assigned to that bin was derived based on the proportion of untreated infection encompassed by that bin. These probabilities were then used to weight random distributions of on-ART proviral sequences into the bins using the random.choices method from the Random module of Python v3.10.9. The random distribution of reservoir sequences into the pre-ART bins was repeated 10,000 times to sample the noise we could expect from this temporally driven model. We then calculated the expected number of sequences in each pre-ART bin by multiplying the bin probability by the total number of unique early on-ART proviral sequences obtained for each individual. A $\chi^{2}$ test statistic was calculated for each set of simulated data by summing the (Observed-Expected)^2^/Expected value for each bin together. This represents the sum of the difference between the observed and expected sequences across all pre-ART bins for a single set of simulated data. For the simulated data, the “observed” value is the simulated number of on-ART proviral sequences in each pre-ART bin, and the expected number of sequences is defined as the bin probability * the number of early on-ART proviral sequences. The $\chi^{2}$ test statistic as described above was calculated for the experimentally derived data, and the proportion of simulated data-derived $\chi^{2}$ test statistics that were greater than or equal to the experimentally-derived $\chi^{2}$ test statistic was determined resulting in a *P*-goodness-of-fit value.

#### Estimating the effect of pre-ART decay

Pre-ART decay was incorporated into the continuous seeding model by determining the number of half-lives that had occurred at each pre-ART time point. This was used to determine what percent of the persistent proviral pool was still present at each pre-ART time point. This percent was then multiplied by the original probability, and the adjusted probabilities were rescaled.

### Estimating participant-specific pre-ART proviral half-life (simplified model of continuous formation only)

On-ART proviral DNA sequences were binned by estimated year of integration. We modified the code used by Brooks et al. to use a negative binomial regression model to estimate each participant’s pre-ART proviral half-life based on the age distribution of on-ART proviral DNA sequences (18). This was done because the assumption that the variance is equal to the mean given by the Poisson generalized linear regression model used by Brooks et al. does not fit this data set as the variance is greater than the mean. Thus, utilizing a Poisson generalized linear regression model would result in over-dispersion. To correct for this, a negative binomial regression model was used. A negative binomial regression model was applied to the grouped counts with the age of the bin (in years) as the predictor. A *χ*^2^ test was used to evaluate the fit of the negative binomial regression model to the data by comparing the residual deviance with the degrees of freedom. For all seven participants, the *P*-value was >0.05.

## Data Availability

The 3' half genome sequences are deposited in GenBank (accession nos. PP295393 to PP295816). Pre-ART RNA sequences were deposited in the Sequence Read Archive (SRA) under accession numbers PRJNA1034561 to PRJNA550394. Code for the continuous formation models with specific decay rates (i.e., simplified model of continuous formation) is available at: https://github.com/ocouncil/Episodic_Reservoir_Formation.git . The source code and counts data implementing the construction and testing of the dUCF and dCFD models of continuous persistent proviral pool formation described above are available in an interactive notebook, at https://observablehq.com/@spond/reservoir-formation-model-testing.

## SUPPLEMENTAL MATERIAL

Fig. S1 jvi.00979-24-s0001.tif Combined viral load graph for the seven women included in this study.

Fig. S2 jvi.00979-24-s0002.tiff Secondary analysis of the data in Fig. 5, pertinent to the issue of decay of the oldest infected cells.

Legend for Fig. S1 jvi.00979-24-s0003.docx Viral load graph for the seven participants. The vertical dashed line represents the time of ART initiation. The horizontal dashed line indicates the assay limit of quantification.

Legend for Fig. S2 jvi.00979-24-s0004.docx Secondary analysis for Fig. 5. Here, we restricted the analysis to individuals for whom the number of on-ART proviral sequences which date to the earliest sampled time point is equal to or greater than the number of sequences expected under the “no decay” model of continuous seeding. By so doing, we are limiting this analysis to individuals for whom we have the strongest evidence that this earliest time point was not underrepresented in our sampling.

Tables S1 and S2 jvi.00979-24-s0005.docx Comparison of early:late ratios in current study compared to previous studies, and list of reagents/primers used.

ASM does not own the copyrights to Supplemental Material that may be linked to, or accessed through, an article. The authors have granted ASM a non-exclusive, world-wide license to publish the Supplemental Material files. Please contact the corresponding author directly for reuse.
