# Supplementary figures and images for: The persistent pool of HIV-1-infected cells is formed episodically during untreated infection
Source: J Virol. 2024 Dec 26;99(2):e00979-24. doi: 10.1128/jvi.00979-24 (PMC11852786; doi:10.1128/jvi.00979-24)

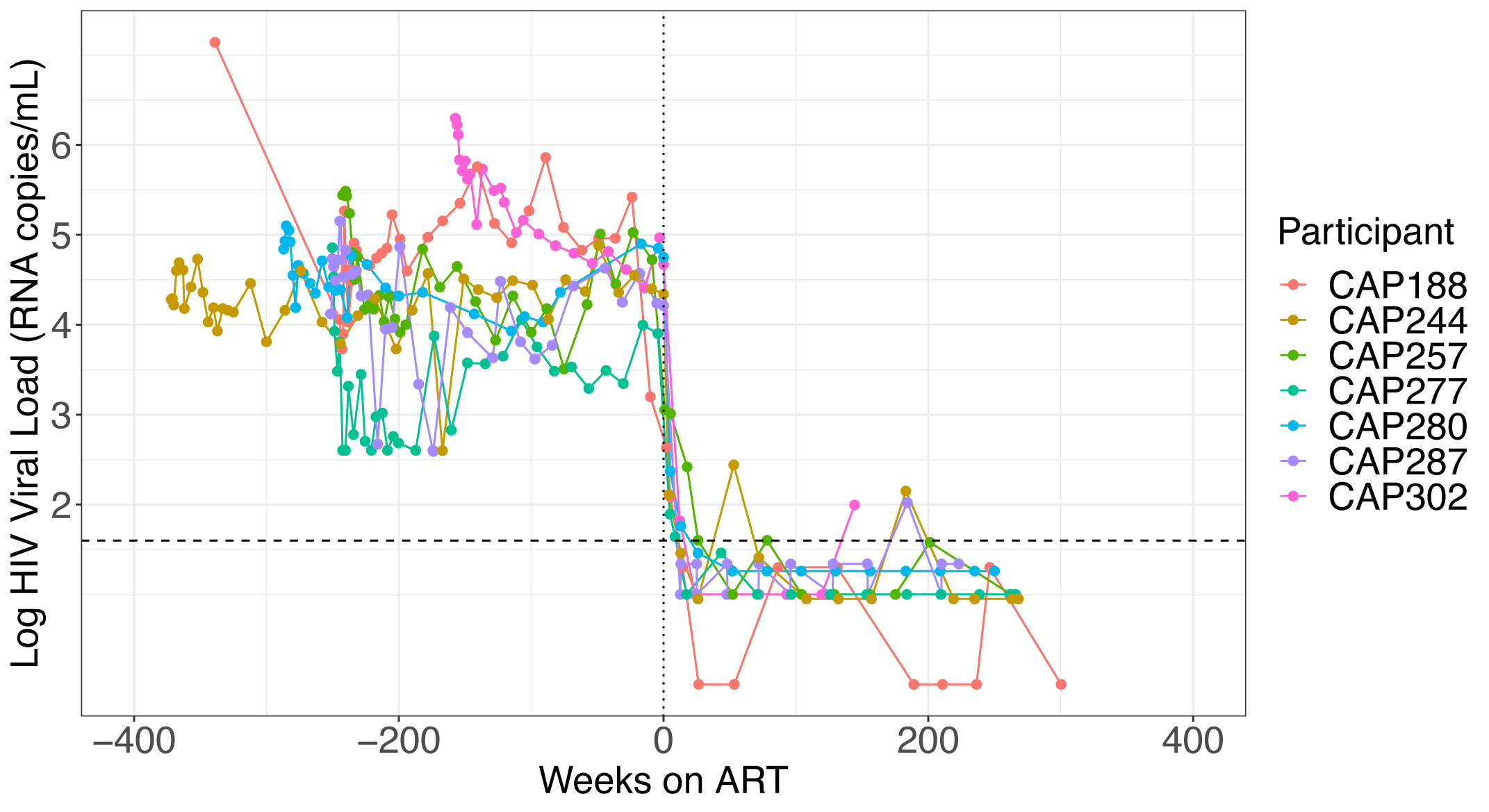

Supplement: Fig. S1 — Combined viral load graph for the seven women included in this study. [file jvi.00979-24-s0001.tif]

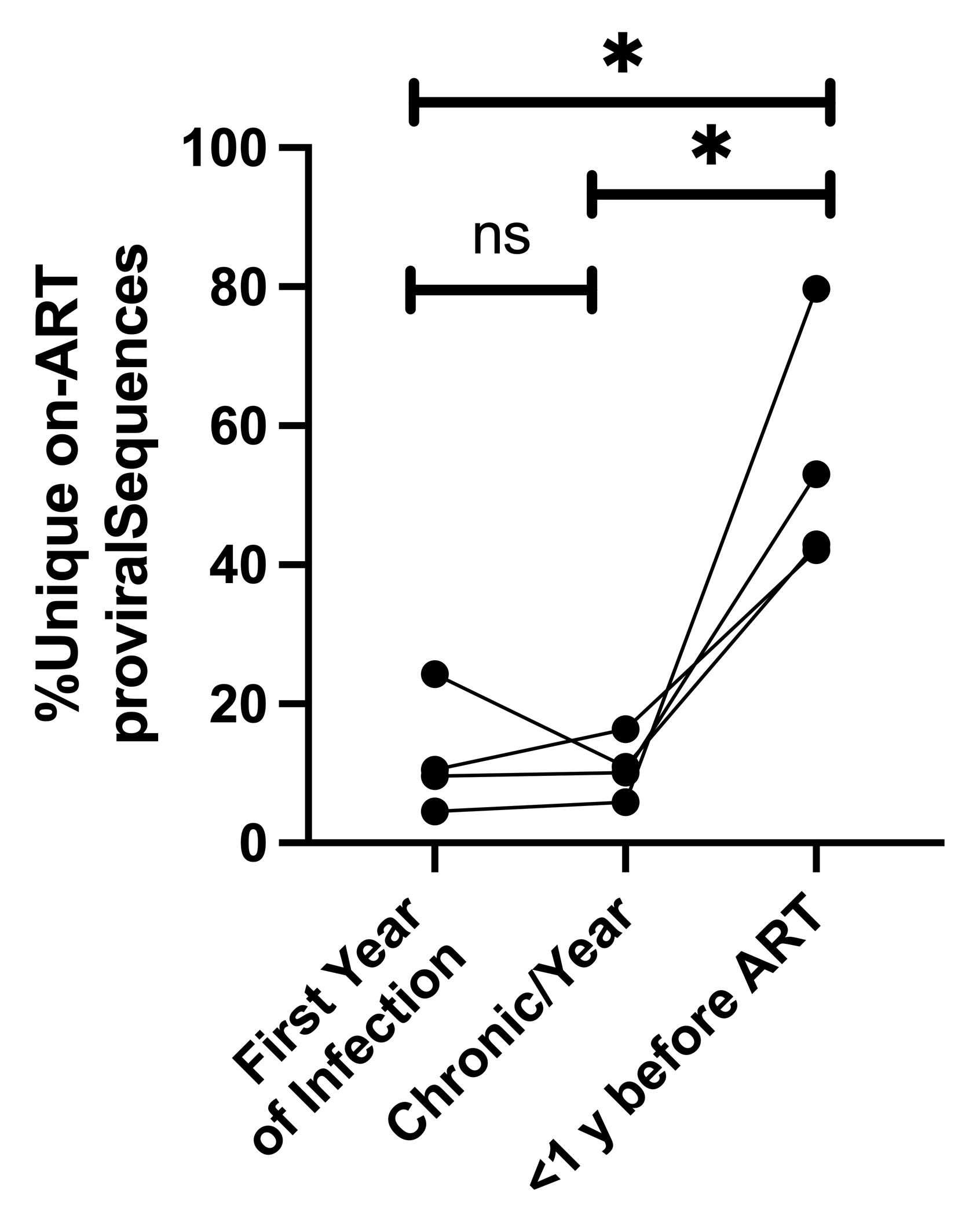

Supplement: Fig. S2 — Secondary analysis of the data in Fig. 5, pertinent to the issue of decay of the oldest infected cells. [file jvi.00979-24-s0002.tiff]
