# Supplementary material for: The persistent pool of HIV-1-infected cells is formed episodically during untreated infection
Source: J Virol. 2024 Dec 26;99(2):e00979-24. doi: 10.1128/jvi.00979-24 (PMC11852786; doi:10.1128/jvi.00979-24)
Supplement: Legend for Fig. S1 — Viral load graph for the seven participants. The vertical dashed line represents the time of ART initiation. The horizontal dashed line indicates the assay limit of quantification. [file jvi.00979-24-s0003.docx]

**Supplemental Figure 1.** Viral load graph during the untreated and treated portions of their infection for each of the seven women whose viral sequences were included in this study.
