# Supplementary material for: The persistent pool of HIV-1-infected cells is formed episodically during untreated infection
Source: J Virol. 2024 Dec 26;99(2):e00979-24. doi: 10.1128/jvi.00979-24 (PMC11852786; doi:10.1128/jvi.00979-24)
Supplement: Legend for Fig. S2 — Secondary analysis for Fig. 5. Here, we restricted the analysis to individuals for whom the number of on-ART proviral sequences which date to the earliest sampled time point is equal to or greater than the number of sequences expected under the “no decay” model of continuous seeding. By so doing, we are limiting this analysis to individuals for whom we have the strongest evidence that this earliest time point was not underrepresented in our sampling. [file jvi.00979-24-s0004.docx]

**Supplemental Figure 2. No increase in persistent proviral pool seeding is observed during acute infection, when analysis is restricted to four most deeply-sampled individuals.** We repeated the analysis shown in Figure 5 on the four individuals for whom the number of unique on-ART proviral sequences that dated to the earliest pre-ART time point was equal to or greater than the expected number of sequences at that time point, under the no decay continouous seeding model. The percent of unique on-ART proviral sequences seeded at each point is shown. Paired t-tests were used to compare the percent of unique on-ART proviral sequences that were seeded at the indicated times. * p < 0.05.
