# Supplementary material for: The persistent pool of HIV-1-infected cells is formed episodically during untreated infection
Source: J Virol. 2024 Dec 26;99(2):e00979-24. doi: 10.1128/jvi.00979-24 (PMC11852786; doi:10.1128/jvi.00979-24)
Supplement: Tables S1 and S2 — Comparison of early:late ratios in current study compared to previous studies, and list of reagents/primers used. [file jvi.00979-24-s0005.docx]

| **Participant** | **No. Late DNA Reservoir Sequences** | | **No. Early DNA Reservoir Sequences** | | **Fisher’s exact p value** |
| --- | --- | --- | --- | --- | --- |
|  | Joseph et al. | This Study | Joseph et al. | This Study |  |
| CAP188 | 14 | 55 | 16 | 61 | >0.9999 |
| CAP244 | 16 | 57 | 14 | 39 | 0.6724 |
| CAP257 | 23 | 106 | 7 | 27 | 0.8037 |
| CAP287 | 16 | 47 | 14 | 61 | 0.4088 |
| CAP277 | 13 | 16 | 17 | 22 | >0.9999 |
| CAP280 | 17 | 44 | 12 | 39 | 0.6682 |
| CAP302 | 14 | 14 | 32 | 32 | >0.9999 |

**Supplemental Table 1.** Fisher’s exact test of the proportion of DNA reservoir sequences seeded “early” vs. “late” for the current study compared to Joseph et al., PLoS Pathogens, 2024.

| REAGENT or RESOURCE | SOURCE | IDENTIFIER |
| --- | --- | --- |
| **Oligonucleotides** | | |
| TCTCGACGCAGGACTCG | Integrated DNA Technologies | LTRgagF |
| TACTGACGCTCTCGCACC | Integrated DNA Technologies | LTRgagR |
| /56-FAM/CTCTCTCCT/ZEN/TCTAGCCTC/3IABkFQ/ | Integrated DNA Technologies | LTRgag_Probe |
| GATTTGGACCTGCGAGCG | Integrated DNA Technologies | RPP30F |
| GCGGCTGTCTCCACAAGT | Integrated DNA Technologies | RPP30R |
| /56-FAM/CTGACCTGA/ZEN/AGGCTCT/3IABkFQ/ | Integrated DNA Technologies | RPPP30_Probe |
| AAATCTCTAGCAGTGGCGCCCGAACAG | Integrated DNA Technologies | U5-623F |
| TGAGGGATCTCTAGTTACCAGAGTC | Integrated DNA Technologies | U5-601R |
| CCCTACAATCCCCAAAGTCAAGGAG | Integrated DNA Technologies | 4653F |
| GCACTCAAGGCAAGCTTTATTGAGGCTTA | Integrated DNA Technologies | OFM19 |
|  |  |  |
| **Software and algorithms** | | |
| GraphPad Prism v9.5.1 | GraphPad Software, LLC |  |
| Python v3.10.9 |  |  |
|  |  |  |
|  |  |  |
|  |  |  |
| **ddPCR reagents** | | |
| DEPC-treated water | Invitrogen | Cat# AM9906 |
| ddPCR Supermix for Probes (no dUTP) | Bio-Rad | Cat# 1863024 |
| DG32 Automated Droplet Generator Cartridges | Bio-Rad | Cat# 1864108 |
| ddPCR 96-well plates | Bio-Rad | Cat# 12001925 |
| Pipet Tips for the Automated Droplet Generator System | Bio-Rad | Cat# 1864120 |
| Foil PCR Plate Heat Sealer, Pierceable | Bio-Rad | Cat# 1814040 |

**Supplemental Table 2. Reagent and primer source**
